# Modulated Electro-Hyperthermia Induces a Prominent Local Stress Response and Growth Inhibition in Mouse Breast Cancer Isografts

**DOI:** 10.3390/cancers13071744

**Published:** 2021-04-06

**Authors:** Csaba András Schvarcz, Lea Danics, Tibor Krenács, Pedro Viana, Rita Béres, Tamás Vancsik, Ákos Nagy, Attila Gyenesei, József Kun, Marko Fonović, Robert Vidmar, Zoltán Benyó, Tamás Kaucsár, Péter Hamar

**Affiliations:** 1 Institute of Translational Medicine, Semmelweis University, 1094 Budapest, Hungary; schvarcz.csaba-andras@med.semmelweis-univ.hu (C.A.S.); danics.lea@med.semmelweis-univ.hu (L.D.); pedro.leroy@phd.semmelweis.hu (P.V.); beres.rita1@med.semmelweis-univ.hu (R.B.); vancsik.tamas@med.semmelweis-univ.hu (T.V.); benyo.zoltan@med.semmelweis-univ.hu (Z.B.); kaucsar.tamas@med.semmelweis-univ.hu (T.K.); 2 1st Department of Pathology and Experimental Cancer Research, Semmelweis University, 1085 Budapest, Hungary; krenacs.tibor@med.semmelweis-univ.hu; 3 Molecular Oncohematology Research Group, 1st Department of Pathology and Experimental Cancer Research, Semmelweis University, 1085 Budapest, Hungary; nagy.akos1@med.semmelweis-univ.hu; 4 Bioinformatics Research Group, Genomics and Bioinformatics Core Facility, János Szentágothai Research Centre, University of Pécs, H-7624 Pécs, Hungary; gyenesei.attila@pte.hu (A.G.); kun.jozsef@pte.hu (J.K.); 5 Department of Pharmacology and Pharmacotherapy, Medical School & Szentágothai Research Centre, Molecular Pharmacology Research Group, Centre for Neuroscience, University of Pécs, H-7624 Pécs, Hungary; 6 Department of Biochemistry and Molecular and Structural Biology, Jožef Stefan Institute, 1000 Ljubljana, Slovenia; marko.fonovic@ijs.si (M.F.); robert.vidmar@ijs.si (R.V.)

**Keywords:** modulated electro-hyperthermia, triple negative breast cancer isograft, tumor growth inhibition, antiproliferative effect, stress response

## Abstract

**Simple Summary:**

Here we investigated the most aggressive type of breast cancer (triple negative breast cancer (TNBC)) for which no effective therapies exist. Modulated electro-hyperthermia (mEHT) utilizes the altered bioelectric properties of tumors to implement a selective energy-transmission of an electromagnetic field and induce thermal and non-thermal anti-tumor effects. In our present study, repeated mEHT treatment effectively inhibited growth and proliferation and caused significant, destruction of TNBC tumors when applied alone without any other therapy in mice. Immunohistochemistry and multiplex analysis revealed that mEHT treatment induced protective mechanisms, like upregulation of heat shock proteins and other stress-related genes. Inhibition of these factors may serve as therapeutic approach to enhance the efficacy of mEHT. We were able to inhibit one of these protective proteins in cell culture. We aim to study the possibility of enhancing mEHT and other cancer therapies by inhibiting the identified protective stress response.

**Abstract:**

Modulated electro-hyperthermia (mEHT) is a selective cancer treatment used in human oncology complementing other therapies. During mEHT, a focused electromagnetic field (EMF) is generated within the tumor inducing cell death by thermal and nonthermal effects. Here we investigated molecular changes elicited by mEHT using multiplex methods in an aggressive, therapy-resistant triple negative breast cancer (TNBC) model. 4T1/4T07 isografts inoculated orthotopically into female BALB/c mice were treated with mEHT three to five times. mEHT induced the upregulation of the stress-related Hsp70 and cleaved caspase-3 proteins, resulting in effective inhibition of tumor growth and proliferation. Several acute stress response proteins, including protease inhibitors, coagulation and heat shock factors, and complement family members, were among the most upregulated treatment-related genes/proteins as revealed by next-generation sequencing (NGS), Nanostring and mass spectrometry (MS). pathway analysis demonstrated that several of these proteins belong to the response to stimulus pathway. Cell culture treatments confirmed that the source of these proteins was the tumor cells. The heat-shock factor inhibitor KRIBB11 reduced mEHT-induced complement factor 4 (C4) mRNA increase. In conclusion, mEHT monotherapy induced tumor growth inhibition and a complex stress response. Inhibition of this stress response is likely to enhance the effectiveness of mEHT and other cancer treatments.

## 1. Introduction

In our previous study we demonstrated that modulated electro-hyperthermia (mEHT) induced Hsp70 upregulation, and exhaustion of this defense mechanism resulted in apoptotic cell death of the mouse 4T1 triple negative breast cancer (TNBC) model. Hsp70 inhibition synergistically enhanced the tumor-killing effect of mEHT [1]. In the present study, our aim was to investigate the molecular effects of mEHT using multiplex methods.

Loco-regional deep hyperthermia (mEHT) is a type of medical hyperthermia usually used as supplementary treatment for cancer patients [2]. mEHT is applied together with conventional modalities like chemo, radio or immunotherapy to enhance their effect and tumor-specificity [3]. mEHT can also boost the effect of targeted therapies, e.g., angiogenesis inhibition by Bevacizumab (Avastin^®^) in different types of malignancies, including breast cancer [4]. Hyperthermia decreases the activity of hypoxia-inducible factor 1 (HIF-1) and contributes to the vascular endothelial growth factor (VEGF)-mediated angiogenesis inhibition of Bevacizumab [5]. mEHT’s potential as a monotherapy is currently under investigation [1,6]. mEHT uses a 13.56 MHz frequency capacitively coupled electromagnetic field that transmits energy to the tumor. mEHT utilizes the bioelectrical difference between the tumor tissue and healthy tissues [7]. The difference in bioelectrical properties results from the higher aerobic glycolysis of cancer cells, known as the Warburg-effect, that causes higher lactate and ion levels in the cancer cells and thus elevates electric conductivity of the tumor [8]. Due to these factors, the energy of the electromagnetic field is absorbed mainly by the tumor.

mEHT can effectively induce caspase-dependent apoptosis as demonstrated by elevated cleaved caspase 3 (cC3) expression in mEHT-treated tumors [1,9]. mEHT induces a heat shock response and subsequent heat shock protein (Hsp70) upregulation in treated tumors [1,10]. In addition, mEHT potently inhibits tumor cell proliferation indicated by the attenuation of Ki67-positive cell nuclei [11,12], a widely used proliferation marker.

Breast cancer is the most frequently occurring cancer type among women worldwide [13]. Fifteen percent of all breast cancers are triple negative, with no hormone—(estrogen, progesterone) and human epidermal growth factor (HER2) receptors on the surface of the cells. Consequently, hormone and anti-HER2 therapies are ineffective and, as TNBC is the most aggressive breast cancer type, prognosis is poor [14]. Thus, complementary therapies are needed to improve the outcome.

The most commonly used mouse TNBC models utilize cell lines derived from mouse mammary carcinoma cell line 410.4 isolated from a single spontaneous tumor in the BALB/c mouse. Cell lines 4T1 and 4T07 are the most aggressive and invasive subclones derived from the 4104 cell line [15,16]. Implantation of these cell lines creates isografts in BALB/c mice. Thus, after the inoculation of syngeneic cells, immune mechanisms can be investigated under conditions very similar to those of human TNBC [17,18].

In the present study we investigated the molecular effects of mEHT using multiplex methods at the RNA (NGS RNA seq, Nanostring) and the protein level (mass spectrometry). The multiplex data revealed that one of the most significant responses to mEHT was the upregulation of acute stress response proteins. These proteins are part of the innate immune system’s nonspecific humoral response as an ancient defense mechanism and have been reported to participate in the immunomodulation of cancer [19], as well as in supporting cancer cells by the formation of extracellular matrix of the tumor microenvironment (protease inhibitors, fibrinogens, haptoglobin, pentraxin). These stress proteins are induced by several forms of tissue injuries, inflammation and in different chronic diseases. They are also often upregulated in cancer patients’ serum [20]. These proteins are regarded collectively as acute phase proteins (APPs). However, according to the generally held concept, the major source of APPs is the liver, and only scarce literature demonstrates local production of these proteins upon tissue injury. However, these factors can be induced by different forms of cell stress, like ischaemia [21] or heat [22], and they can contribute to disease elimination and the restoration of homeostatic conditions via different mechanisms [23,24]. The local production of these stress proteins (protease inhibitors, coagulation and complement factors, as well as heat shock proteins) in tumors has been reported to contribute to tumor progression by supporting carcinogenesis, tumor growth, proliferation and angiogenesis [25], and their elevation is considered as a poor prognostic factor [20]. Therefore, inhibition of some of these stress-response proteins such as heat shock proteins [1,9], complement [26], haptoglobin [27] or fibrinogen [28], has recently been proposed as a promising new direction for cancer treatment. However, this is the first report comprehensively demonstrating the local production of several stress-induced proteins in TNBC as a defense to treatment, using multiplex methods. Furthermore, we demonstrate that the specific heat shock inhibitor KRIBB11 abrogates complement upregulation (C4). Thus, our data support that besides inhibition of the heat-shock response, complement inhibition may be utilized in cancer therapy and may synergistically improve the therapeutic effectiveness of mEHT.

## 2. Results

### 2.1. mEHT Reduced Tumor Growth

**Figure 1:**
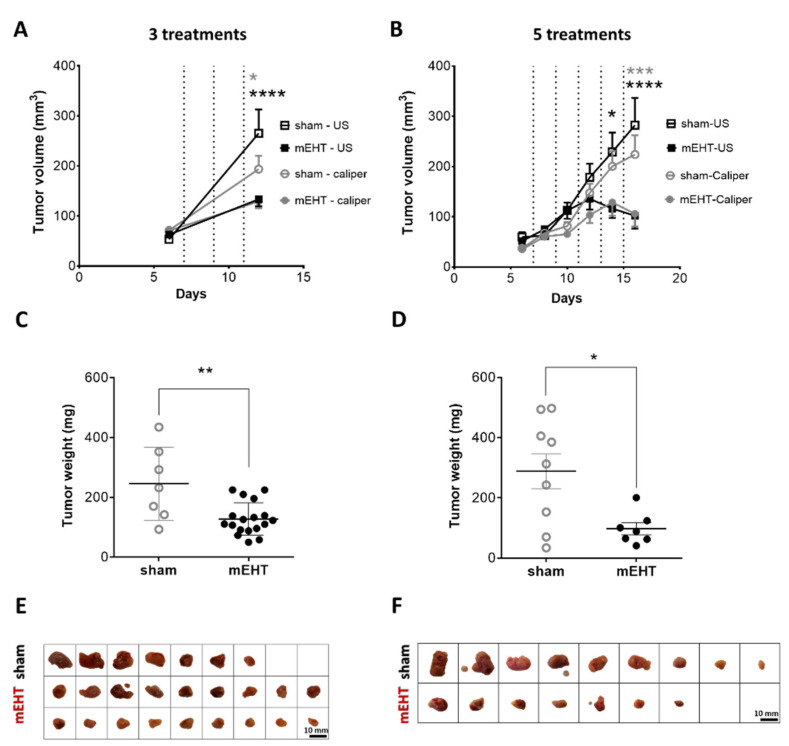
Effect of repeated modulated electro-hyperthermia (mEHT) treatments on tumor size and weight. Digital caliper and ultrasound data after three (**A**,**C**,**E**) and five (**B**,**D**,**F**) treatments (dotted lines, **A**,**B**). Tumor weight (**C**,**D**), scale images of the excised tumors (**E**,**F**). (**A**–**E**) *n*_(sham)_ = 7, *n*_(mEHT)_ = 18. (**B**–**F**) *n*_(sham)_ = 9, *n*_(mEHT)_ = 7. Mean ± SEM, (**A**,**B**) two-way ANOVA, Bonferroni correction, (**C**,**D**) Mann-Whitney test, *: *p* < 0.05, **: *p* < 0.01, ***: *p* < 0.001, ****: *p* < 0.0001. Cell line: 4T07, 3–5× treated.

Follow-up measurements of the tumors by ultrasound (US) and a digital caliper demonstrated a decline in the tumor growth rate in the mEHT-treated group (Figure 1) Tumor volume significantly decreased after three treatments in the mEHT-treated group as measured both by caliper (*p* = 0.0363) and US (*p* < 0.0001) (Figure 1A). Further (4–5) treatments were able to reduce tumor size. The difference in volume became even larger after five treatments (US: *p* < 0.0001, caliper: *p* = 0.0003) (Figure 1B). Comparison of the final and initial tumor volumes demonstrated a higher growth ratio in sham-treated (three treatments: US: 5.0, caliper: 2.7; 5 treatments: US: 4.7, caliper: 6.0), than in mEHT-treated (three treatments: US: 2.1, caliper: 1.8; 5 treatments: US: 2.0, caliper: 3.0) tumors. The weights of the excised tumors were also significantly smaller in the mEHT-treated than in the sham group after three (*p* = 0.0091) and five treatments (*p* = 0.0206) (Figure 1C,D). The excised tumors were significantly smaller in the mEHT-treated versus sham-treated mice (Figure 1E,F). After five treatments in one mEHT-treated mouse, ultrasound demonstrated a small tissue at the site of the tumor. However, by dissection and histological analysis, this tissue was shown to contain no tumor cells, only fat and connective tissue. Therefore, this tumor was regarded as treated (Figure 1F) and its data were included in the tumor growth (Figure 1B) but not in the tumor weight (Figure 1D) data.

### 2.2. mEHT Induced Caspase-3-Positive Tumor Tissue Destruction

**Figure 2:**
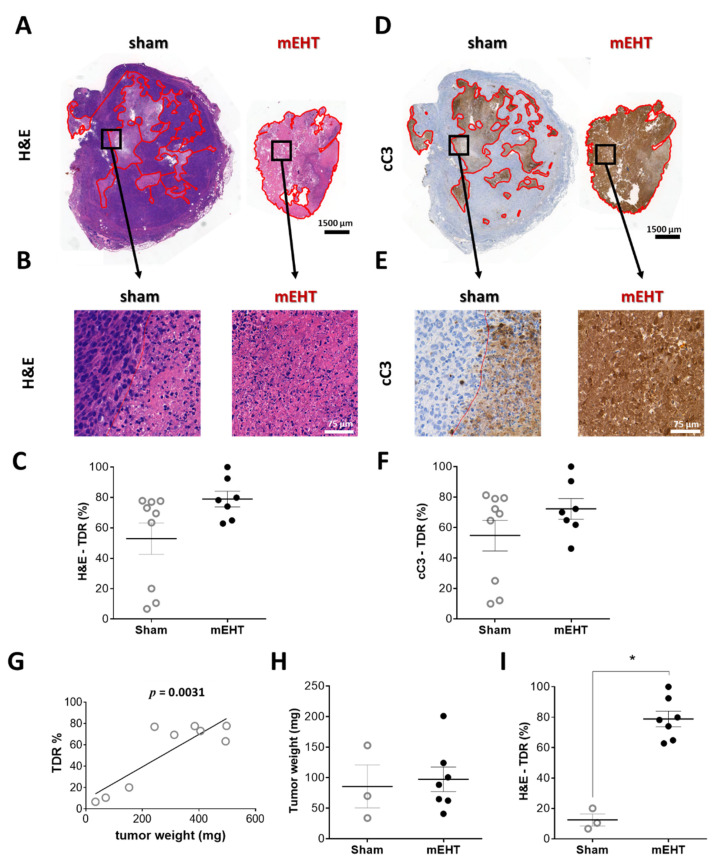
Effect of mEHT treatment on tumor destruction ratio (TDR) in hematoxylin-eosin-(H&E)-stained, and cleaved caspase-3 (cC3) immunohistochemistry-stained sections 24 h after the 5th mEHT treatment. Representative H&E-stained tumors from sham and mEHT-treated groups with 0.9× (**A**) and 40× (**B**) magnification. Destructed area is annotated (red). TDR (%) evaluated on H&E (**A**–**C**) and cC3 (**D**–**F**) sections. Representative cC3 (**D**,**E**) stained tumors with low (0.9×, **D**) and high (40×, **E**) magnification. Correlation between tumor weight and TDR (%) in sham animals (**G**). Three smallest sham tumors with weights similar to those of mEHT tumors (**H**). Comparison of TDR (%) of sham and mEHT tumors of similar weight (**I**). Mean ± SEM, Mann-Whitney test, *n* = 7–9/group, *: *p* < 0.05. Cell line: 4T07, 5× treated.

The tumor destruction ratio (TDR), evaluated in hematoxylin and eosin (H&E) sections, demonstrated remarkable tumor tissue destruction in the mEHT-treated tumors (TDR: mEHT: 78.9 ± 5.1% vs. sham: 52.8 ± 10.3%) (Figure 2A,B). Besides the three small tumors in the sham group, TDR was not significantly smaller in the sham-treated animals than in the mEHT group (Figure 2C). The destroyed area identified on the H&E sections stained cleaved caspase-3 (cC3) positive on consecutive cC3-stained sections, confirming a caspase-dependent manner of tumor destruction (Figure 2D–F). The complete set of all tumors (H&E and cC3 staining) is presented in the Supplementary Material (Figure S1). 410.4-derived tumors developed a necrotic core after reaching a certain size [29]. In the present study TDR strongly correlated with tumor size (Figure 2G). Comparison of TDR in tumors of similar size (sham vs. mEHT, Figure 2H) demonstrated a significant increase (*p* = 0.0167) in TDR by mEHT treatments, corroborating that in the sham group TDR elevation was size-dependent, whereas in the mEHT group it was size-independent but treatment-related (Figure 2I).

### 2.3. mEHT Induced Heat Shock Protein 70 (Hsp70) Accumulation

**Figure 3:**
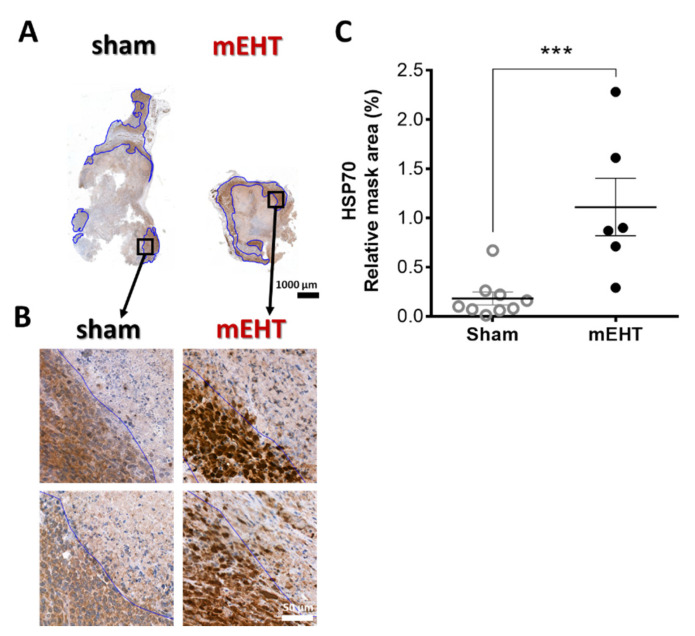
Effect of mEHT treatment on Hsp70 expression 24h after the 5th mEHT treatment. Representative tumors from sham and mEHT-treated mice with Hsp70 staining (0.9× magnification), black rectangles magnified at ‘B’ (**A**). Representative sections of Hsp70 expression near the damaged tumor area (blue annotation), 40× magnification (**B**). Expression of Hsp70 evaluated in the intact tumor tissue (blue annotations) significantly increased in mEHT-treated tumors (**C**). In one case, TDR appeared to be 100% and no living area remained to be evaluated for Hsp70 expression. Mean ± SEM, Mann-Whitney test, *n* = 6–9/group, ***: *p* < 0.001. Cell line: 4T07, 5× treated.

Specific Hsp70 immunostaining in mEHT-treated animals was intense brown (DAB) in the living cells around the damaged core area of the tumor. Such intense (specific) staining was absent in sham-treated tumors (*p* = 0.0008) (Figure 3A–C).

### 2.4. mEHT Reduced Ki67 Expression

**Figure 4:**
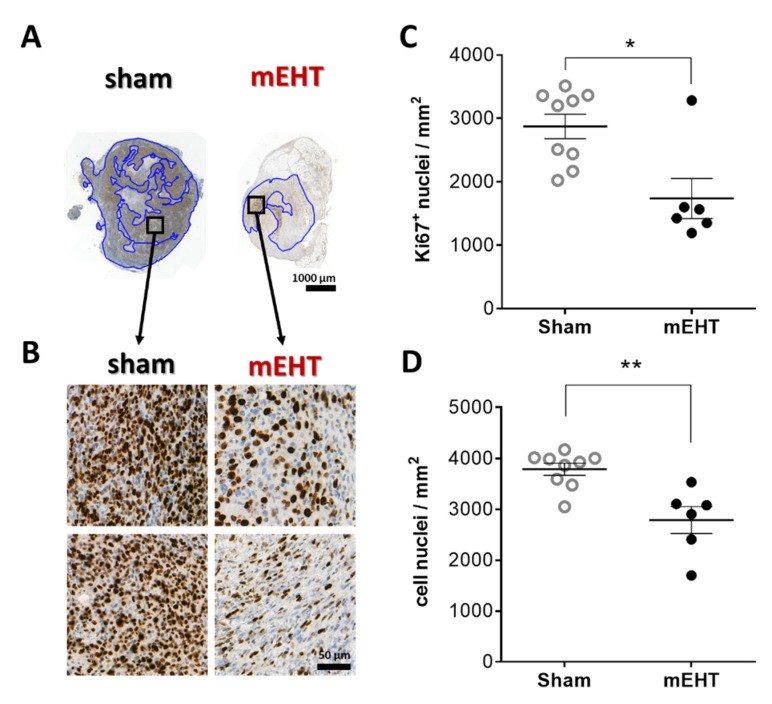
Effect of mEHT treatment on Ki67 expression 24h after the 5th mEHT treatment. Representative tumor sections from sham and mEHT-treated mice with Ki67 staining (**A**,**B**). Blue annotations: intact area where Ki67^+^ nuclei were evaluated (0.9× magnification, black rectangles magnified at ‘B’ (**A**), 40× magnification (**B**)). Area-proportional number of strongly Ki67 positive (**C**) and all nuclei in the intact tumor area (**D**). Mean ± SEM, Mann-Whitney test, *n* = 6–9/group, *: *p* < 0.05, **: *p* < 0.01. Cell line: 4T07, 5× treated.

Most cell nuclei in sham tumors were intensely positive for the Ki67 proliferation marker in the living tumor area (Figure 4A,B). mEHT treatment significantly decreased Ki67 positivity (*p* = 0.0120) (Figure 4C). The number of total cell nuclei counted in the living tumor area was also significantly lower in the mEHT-treated compared to the sham-treated group, with a significantly less dense tissue structure due to mEHT treatments (*p* = 0.0048) (Figure 4D).

### 2.5. Multiplex Analysis of mEHT Effects on Gene Expression

**Figure 5:**
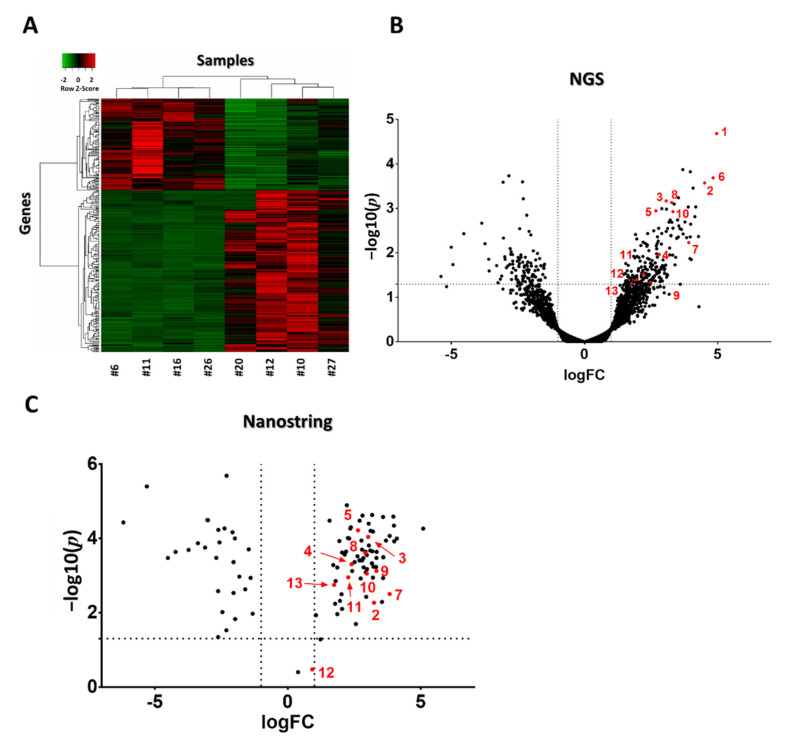
Heat map and volcano plot visualization of DE genes after three mEHT treatments. Heat map clusterization with dendograms (Kendall tau’s method, dendogram details: Figure S2) of differentially expressed (DE) genes according to the next generation sequencing (NGS) RNA seq data. Columns represent samples, rows represent genes. Red = upregulation, green = downregulation (**A**). Volcano plot of all genes according to the NGS RNA seq data (**B**). Volcano plot of DE genes from the Nanostring data (**C**). (**B**,**C**) −log10(*p*) values plotted against fold changes (logFC). Vertical dotted line: logFC = 1, horizontal dotted line: −log10(*p*) = 1.30103. *n* = 4–6/group. Red dots with numbers mark genes identified in Table 1. Cell line: 4T1, 3× treated.

Next-generation sequencing of RNA (NGS RNA Seq) was performed from 4T1 tumor samples 24 h after the third mEHT treatment to investigate gene changes induced by mEHT. Two hundred ninety genes were differentially expressed (DE, criteria: *p* < 0.05 or log10(*p*) < 1.30103; Fold Change (FC) > 2 or logFC > 1). Heat map visualization clustered with Kendall’s Tau distance measurement clearly showed that the vast majority of DE genes were upregulated due to the treatments: one hundred eighty-five upregulated and one hundred five downregulated genes appeared (Figure 5A) A dendogram labeled with gene names is presented in our Supplementary Material (Figure S2). A Volcano plot visualization of gene logFC and –log10(*p*) values is presented in Figure 5B. For validation of gene expression at the mRNA level, individual mRNA molecular counting was performed with Nanostring nCounter^®^ Technology (Nanostring Technologies, Seattle, WA, USA). One hundred and thirty-four DE target genes from NGS data were sorted to create a custom Nanostring gene panel. One hundred and four target genes were identified with Nanostring. All of the target genes’ direction of change (up or downregulation) was the same as that detected by NGS. Three genes didn’t fulfill the DE criteria (Figure 5C).

**Figure 6:**
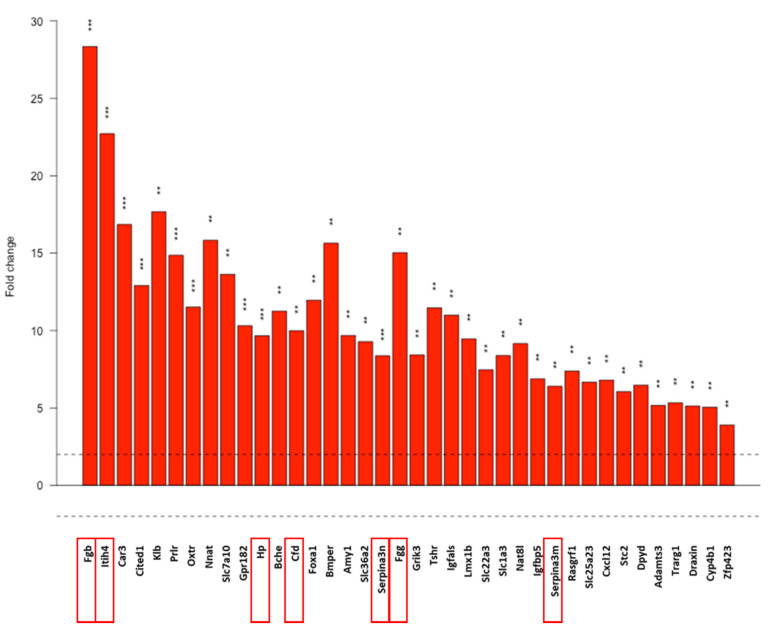
Response to stimulus pathway based on the gene-ontology (GO) analysis of the NGS data. Dotted line: FC = 2. Red frames: further analyed genes. Gene names, *p* values and further pathways containing stress-related genes analyzed with the DEListEnrichment_upR module are presented in Table S2. **: *p* < 0.01, ***: *p* < 0.001. Cell line: 4T1, 3× treated.

We focused on the upregulated DE genes. These genes were sorted into nineteen functional categories created by us, based on a literature search. Gene ontology (GO) pathway analysis of upregulated genes (DEListEnrichment_upR) revealed that most upregulated genes (38 genes) clustered into the response to stimulus pathway (GO:0050896; pathway *p* value: 0.00012, Figure 6). Tabular display of the pathway is presented in our Supplementary Material (Table S1). Various types of stress-related genes (coagulation factors, protease inhibitors, complement factors) are included in this pathway. Therefore, we investigated these genes further.

mEHT treatment induced innate immune-response reactions, among others, in the tumor. Thirteen stress-related genes were observed to be significantly upregulated, including protease inhibitors (Itih2, Itih4, Serpina3n, Serpina3c, Serpina3c), coagulation factors (Fbg, Fgg), the free hemoglobin-binding haptoglobin (Hp), and complement cascade-related genes including secreted pattern recognition receptor (PRR), pentraxin-related protein 3 gene (Ptx3), classical pathway (C1s1, C4b), alternative pathway (Cfd) and terminal pathway (Hc) complement components. Fold-changes and *p* values of the aforementioned significantly upregulated genes, detected with NGS RNA Seq and Nanostring, are presented in Table 2. To investigate if APP upregulation appears not just at the mRNA but also at the protein level, mass spectrometry (MS) examination was performed fromwith the same samples. Eight out of thirteen APPs detected with NGS also appeared at the protein level, and demonstrated significantly upregulated levels when examined by MS. Four heat shock proteins were detected as significantly upregulated by MS, but not by NGS. Label-free quantification intensity difference (LFQ Int. Diff.) values of the given proteins detected by MS are shown in Table 2.

Nanostring data provided absolute RNA count cellular stress response factors (Table 1). These data further oriented our research, since those targets that demonstrated low absolute expression despite fulfilling DE criteria (*p* < 0.05, FC > 2) were excluded from further investigation. Thus, we focused on Serpina3n, haptoglobin (Hp), pentraxin (Ptx)3, and the complement factors (Cfd, C4b) with high absolute RNA counts and high FC without overlapping values between the groups (bold letters in Table 1).

### 2.6. The Heat-Shock Factor-1 Inhibitor KRIBB11 Reduced C4b Expression In Vitro

**Figure 7:**
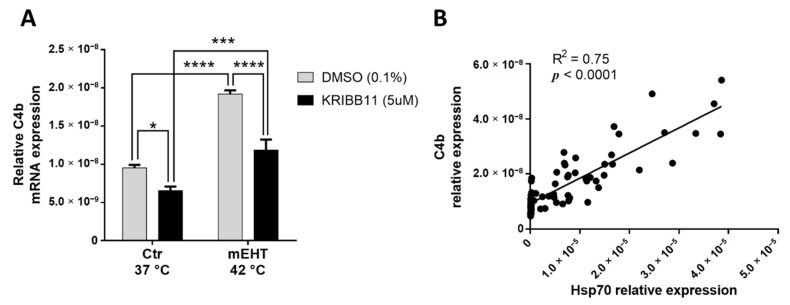
KRIBB11 effect on mEHT-induced C4b production. C4b mRNA relative expression, 2 h post-mEHT, normalized to 18S, with KRIBB11 treatment, vs. DMSO (**A**). Correlation between C4b and Hsp70 expression (**B**). Mean ± SEM, Two-way ANOVA, *n* = 5–15/group, *: *p* < 0.05, ***: *p* < 0.001, ****: *p* < 0.0001. Cell line: 4T1.

One of the most upregulated genes/proteins with significant upregulation on all three screens was the mouse C4 complement C4b. C4b mRNA was measurable by qPCR from in vitro 4T1 cell culture, demonstrating that 4T1 tumor cells produce C4b. mEHT treatment in monotherapy induced a significant upregulation of C4b mRNA 2 h after treatment in vitro (*p* < 0.0001; Figure 7A). We demonstrated in our earlier paper [1] that KRIBB11 (N(2)-(1H-indazole-5-yl)-N(6)-methyl-3-nitropyridine-2,6-diamine) reduced the cellular heat shock response of 4T1 cancer cells through inhibiting the heat-shock factor (Hsf)-1. Therefore, we hypothesized that heat shock-related complement production can be targeted by KRIBB11. KRIBB11 significantly reduced baseline C4b mRNA compared to Dimethyl sulfoxide (DMSO)–treated cells in the 37 °C control group (*p* = 0.0256; Figure 7A). After mEHT treatment, the mEHT-induced C4b elevation was also significantly inhibited by KRIBB11 compared to DMSO treatment (*p* < 0.0001; Figure 7A). Moreover, C4b correlated significantly with Hsp70 expression (*p* < 0.0001; Figure 7B)

## 3. Discussion

In our previous paper [1] we presented the LabEHY200 treatment apparatus with newly developed electrodes, able to perform selective mEHT treatment in a TNBC mouse model. It clearly demonstrated the antitumoral effects of mEHT, which resulted in elevated tumor tissue destruction and reduced cell viability in vivo, even after a short-term protocol (1× or 2× mEHT). Our current paper describes the long-term effects of repeated mEHT treatments on tumor progression in a triple negative mouse breast cancer model for the first time. This is the first comprehensive, multiplex analysis-based investigation of the overall anticancer effects of mEHT at both the gene and protein level. Here, we report that mEHT, even in monotherapy, was able to reduce the growth rate of the highly aggressive triple negative 4T07 isografts. In the background of this strong tumor-inhibitory effect of mEHT we observed reduced proliferation of tumor cells, and heat shock-induced caspase-mediated tumor tissue damage. Multiplex analysis of the mEHT-treated 4T1 tumors revealed massive, local upregulation of protease inhibitors and coagulation and complement factors as a response to cellular stress. These factors are part of the innate immune system’s acute phase reaction. However, being produced locally in tumors, they have a more complex role, depending on the tumor microenvironment and cellular source [23].

Tumor growth was significantly inhibited by mEHT as demonstrated by digital caliper and ultrasound measurements and confirmed by the dramatically smaller weights of the excised tumors in mEHT-treated mice. Moreover, mEHT-treated tumors began to shrink only after the fifth treatment. Tumor shrinkage was not observed after only two treatments, despite a significant induction of tumor cell damage [1]. The tumor growth inhibitory effects of mEHT monotherapy in other tumors were quite similar in previously published studies.: A single (colorectal cancer (CT26) isografts [30,31]) or three (squamous cell carcinoma (SCCVII) isografts [32]) mEHT treatments induced slower tumor growth, but no decline in tumor volume. In our experimental design we applied noninvasive temperature detection during the experiments. Animals were randomized based on tumor size (Table S1) before treatment initiation, overcoming some shortagescomings of previous experiments [30,31]. Similar to our study, five mEHT treatments induced a measurable tumor shrinkage of HepG2 hepatic cancer xenografts [33] and U87-MG rat glioma xenografts [34]. One possible explanation for the lack of measurable tumor shrinkage after three treatments, despite obvious tumor destruction, is a delayed clearance of the apoptotic tumor cells. APPs have a major role in apoptosis, e.g., Pentraxin 3 (PTX3) binds to apoptotic cells and facilitate their clearing by macrophages via the Fcγ receptor [35] and dendritic cells [36]. An immunologically silent clearance of apoptotic tumor cells was reported to be mediated by an opsonizing effect of complement factors [37]. Thus, the detected upregulation of PTX3 and C4 may contribute to the clearance of apoptotic cancer cells and tumor shrinkage after five treatments.

As we demonstrated in our previous paper [1], Hsp70 is a reliable marker of mEHT treatment effects and, similar to two treatments, in our current paper five mEHT treatments resulted in strong upregulation of Hsp70. The MS study demonstrated the upregulation of several heat shock proteins (Hspβ1, Hsp70-1A, Hsp70-1B, Hsp105), corroborating our IHC data. These proteins were also detected at the RNA level by NGS, but there was no significant difference between the two groups. This finding is in accordance with our previous findings, demonstrating that 24 h after mEHT treatment the Hsp70 response returns to baseline at the mRNA level but not at the protein level [1].

In this study we observed a significant tumor size reduction by mEHT treatment, but the damaged area (tumor destruction ratio, TDR) in the mEHT-treated tumors did not differ significantly from the TDR in large sham-treated tumors. The explanation for the extensive destruction in sham tumors is spontaneous necrosis due to their large size. Spontaneous necrosis, due to low oxygen and nutrient supply, is well-known in fast progressing tumors such as the 4T1/4T07 [38,39,40,41]. Consequently, the degree of destruction is consistent with tumor size [42,43,44]. As sham tumors in our study were very large, a central necrosis developed. In contrast, mEHT-treated tumors were much smaller, but their TDR was comparable to that of sham tumors. This implies that the increase of tumor tissue destruction was only size-related in the sham group, but treatment-related in the mEHT group.

Furthermore, mEHT was able to diminish Ki67+ proliferation of this highly proliferative tumor. Ki67 is strongly correlated with aggressiveness and worse prognosis, especially in breast carcinomas, where it is a prognostic marker and one of the molecular features of disease classification [45,46,47]. In our study, not only the proliferative activity, but also the cell density of the tumor tissue was reduced. Only one laboratory reported similar results after a single mEHT treatment of C26 colorectal cancer isografts, but they detected loss of Ki67 expression in the already damaged and early apoptotic areas, while the living tumor around the destructed area seemed to be strongly Ki67-positive [11]. In the present study we demonstrated the loss of proliferating activity of viable tumor cells, which may have led to the diminished tumor growth rate in the mEHT-treated mice.

mEHT activated the local production of several stress-related factors (protease inhibitors: Itih2, Itih4, Serpina3n, Serpina3c, Serpina3m, coagulation related factors: Fgb, Fgg, Hp, and complement related factors (Cfd, C4b, Hc, C1s1, Ptx3)) both at the mRNA and protein level. In fact, induction of these proteins was most significant, with the highest fold-change values in our multiplex next generation sequencing (NGS RNA seq), Nanostring and mass spectrometry (MS) study. Despite the different methods, fold-change mRNA values were very similar with NGS and nanostring, demonstrating good reliability of these methods. A great advantage of the nanostring method is that it detects the absolute copy number of the RNA molecules directly, without reverse transcription and amplification. Moreover, gene ontology (GO) analysis revealed that the most differentially expressed (DE) upregulated genes were related to the response to stimulus pathway (GO:0050896). Thus, in our further studies, we focused on the abundant genes (high RNA copy number) with significant induction by mEHT (no overlap in RNA copy number and FC > 4). The lack of detection of some genes with mass spectrometry may be due to the time lapse between mRNA (as detected by NGS, nanostring) and protein (MS) expression. Here we would like to emphasize that we detected messenger RNA and protein from the tumor tissue, indicating local production of these stress related proteins by the tumor, contrary to the general view about the liver-driven acute phase response [48]. Extra-hepatic synthesis of these proteins has been documented before [49]. Often these genes are regulated by mechanisms different from those acting in hepatocytes [50]. Production of these proteins as a cellular stress-response has been demonstrated before by us [21] and others [51,52], and in the case of cancer cells [53,54]. Similar to our results, photodynamic therapy of fibrosarcoma induced production (mRNA) of other acute phase proteins (serum amyloid-P (SAP) mannose binding lectin (MBL) and c-reactive protein (CRP)) [53]. A recent review demonstrated association of different patterns of acute phase protein production with various types of cancers [54]. A possible interpretation of this finding is that the different patterns are due to protein production by the cancer itself, and not by the liver.

The genes we found massively upregulated by mEHT are all related to cellular stress response and appear to have a general, tumor-protective role in different types of cancer.

The protease inhibitors (serpins and ITIHs) have been described as ancient markers of cell stress [55]. Serine protease inhibitor family A member 3 (Serpina3) was reported as an antiapoptotic factor in trophoblast cells [56]. Furthermore, high expression of Serpina3 was reported in colon [57] and endometrial [58] cancers, and in melanoma [59]. Serpina3n was described as a cellular stress response gene in different types of stress and has been associated with a wide range of diseases such as photoreceptor cell loss in a retinal degeneration mouse model [60], and muscle atrophy in mice and humans [61]. The other members of these protease inhibitors detected, namely Itih2,4 and SerpinA3c,m, had very low absolute mRNA copy numbers, especially in the sham animals, hardly exceeding background values. Even in the mEHT-treated animals, copy numbers were low. Thus, we did not investigate these factors further.

The association of coagulation factors and cancer was first described in 1865 [62]. Fibrinogen (especially Fgg) production by breast cancer cells has been demonstrated before [63]. The production of fibrinogen without fibrin formation contributed to extracellular matrix (ECM) production in breast cancer [64]. Fibrin(ogen) surrounding tumor cells may protect them by acting as a barrier [65]. Thus, fibrinogen synthesized by tumor cells promoted tumor growth [63]. Inhibition of fibrinogen (Fgg) production reduced chemotherapy resistance [28] and growth [63] of breast cancer. Thus, inhibition of mEHT-induced fibrinogen upregulation may have tumor inhibitory effects through diminishing the supportive tumor microenvironment and could synergize with mEHT.

Taken together, the upregulation of both protease inhibitors and fibrinogens seem to contribute to stability of the tumor microenvironment (TME). Thus, the inhibition of these proteins may aid several cancer-treatment forms by inhibiting the formation of a protective tumor microenvironment and facilitating the access of the therapy to the tumor cells.

The primary role of haptoglobin (Hp) is the binding of free Hb released by erythrocytes upon hemolysis. As cell-free hemoglobin is an oxidant, Hp protects from oxidative stress [66]. However, in breast cancer, the Hp mRNA level was significantly higher in the tumor tissue compared to normal breast tissue. Hp production was also reported to be tumor promoting by inducing glycolysis, whereas Hp inhibition by siRNA was antiproliferative and reduced tumor size [27]. Thus, Hp inhibition should be antiproliferative and could synergize with mEHT.

The complement system has been considered for a long time as a simple lytic cascade, aimed at killing bacteria. Nowadays, it is well accepted that complement is a complex innate immune surveillance system, playing a key role in host homeostasis, inflammation, and defense [37]. In the tumor microenvironment, complement factors can perform nocanonical functions [23], such as stimulation of angiogenesis, inflammation, proliferation and migration, and they can even attenuate immunotherapy [25,67]. The therapeutic inhibition of complement components for cancer treatment has been well described [26], and the angogenesis-stimulating role of complements advocates the concept of applying mEHT in combination with angiogenesis inhibitors like Bevacizumab, which has been demonstrated to be beneficial for patients [4]. In our multiplex studies of isolated tumor tissue, the mouse complement C4 (C4b) gene was detected as one of the most upregulated genes/proteins with all three methods (NGS, Nanostring, MS). The complement factor C4 has two isotypes encoded by the C4A and C4B genes in humans, as well as in mice (C4a, C4b). The C4A/C4a (Rodgers blood group) gene encodes the acidic form, whereas the C4B/C4b (Chido blood group) encodes the basic form. Their role in the complement cascade is identical [68]. Production of C4 by nonhepatic (endothelial cells, fibroblasts) cells in the TME has been well described [23,69]. However, we detected C4 in cultured 4T1 cells, demonstrating that C4 was produced by the tumor cells themselves. Production of C4 by 4T1 cells has not been described before. mEHT treatment further upregulated the production of C4, 2 h after treatment in vitro, corroborating our in vivo multiplex data. As mEHT induced a heat shock response, as demonstrated by Hsp70 upregulation, inhibition of HSR by the specific heat-shock factor-1 inhibitor KRIBB11 synergized with mEHT, as demonstrated in our previous paper. Here, we demonstrated that KRIBB11 significantly decreased C4 mRNA. This is a newly described effect of KRIBB11. C4 has been reported to be important for the growth of cervical (TC-1) tumors [70]. Moreover, serum C4 levels may have a prognostic value [71] correlating with tumor size [72]. Finally, inhibition of C4 along with VEGFA inhibition inhibited tumor progression [73]. C4 may act by activating C3 and C5 into their active forms. Furthermore, the alpha chain may be cleaved to release C4 anaphylatoxin [37]. Taken together, the C4-inhibiting effects of KRIBB11 may be beneficial in anticancer therapies and can synergize with mEHT in clinical practice.

Pentraxin 3 (PTX3) is another ancient molecule involved in various cell stress responses, such as oxidative stress [74], a key player in the innate immunity involved in inflammatory responses and wound healing and is a component of the extra-cellular matrix (ECM). Most cell types, including tumor cells, are able to produce PTX3. The PTX3 interactome includes complement [75] and ECM components and apoptotic cells [76]. In breast cancer, PTX3 was induced by hypoxia and correlated with poor prognosis, inducing stem-cell-like characteristics and metastasis formation [76]. Although, antitumoral effects have been also reported, overexpression of PTX3 accelerated metastasis [77], whereas knockdown suppressed tumorigenicity [78]. Thus, PTX3 inhibition may synergize with anti-tumor therapies, including mEHT.

In conclusion, modulated electro hyperthermia (mEHT) has effective antitumor effects, even in monotherapy, in our highly aggressive and rapidly growing 4T1/4T07 triple negative breast cancer in vivo mouse model. The mEHT-induced significant tissue stress was indicated by the upregulation of Hsp70 and cleaved/activated caspase-3, and by the local production of other ancient stress response proteins. The exhaustion of these protective mechanisms resulted in diminished cancer proliferation and caspase-mediated apoptotic tumor cell death. Inhibition of the protective heat shock response and complement C4 production by a specific heat-shock factor inhibitor, KRIBB11, suggests that inhibitors of such stress response proteins may synergize with antitumor therapies such as mEHT.

## 4. Materials and Methods

### 4.1. Tumor Model

410.4 cell-line derived triple negative murine breast cancer cells (4T1/4T07) were grown in cell culture and processed for inoculation as described previously by Ostrand-Rosenberg et al. [79]. Previously [1], we demonstrated significant tumor inhibition after 2 mEHT treatments enhanced by simultaneous inhibition of the heat-shock response by KRIBB11. In the present studies we investigated the proteomic response after three treatments and long-term effects on tumor progression after five treatments. The experiments investigating mEHT effects on tumor progression after three or five treatments were performed on the more immunogenic 4T07 tumors. However, the multiplex and in vitro studies were performed from the more commonly used and more aggressive 4T1 cell line to obtain results which are more generally applicable.

**Figure 8:**
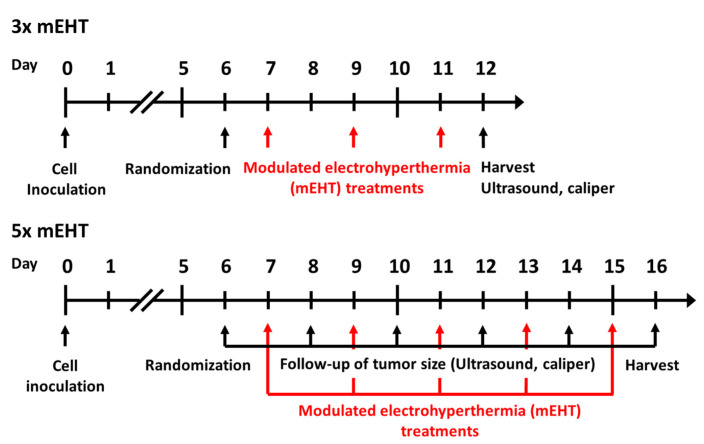
Timeline of in vivo experiment protocols. Cell inoculation was performed at day zero, randomization at day six in both short- and long- term experiments. Modulated electrohyperthermia treatments were performed at day 7, 9, 11 in the short and at day 7, 9, 11, 13, 15 in the long-term experiment. Ultrasound, caliper measurements were performed at day 6 and 12 in the short-term, and at day 6, 8, 10, 12, 14, 16 in the long-term experiment. Harvests were performed in the short- and long-term experiments at day 12 and 16, respectively.

Six- to eight-week-old female BALB/c mice were kept under 12 h dark/light cycles with ad libitum access to food and water in the animal department of Basic Medical Center, Semmelweis University. For tumor-cell inoculation, animals were narcotized with isoflurane (Baxter International Inc., Deerfield, IL, USA), 4–5% for induction, 1.5–2% to maintain anesthesia, with compressed air (0.4–0.6 L/min). Cells were inoculated in standard, 1 × 10^6^ cells /50 μL PBS (Phosphate Buffered Saline without Calcium and Magnesium #17-516F, Lonza A. G., Basel, Switzerland). Inoculations were performed subcutaneously by a Hamilton syringe (Hamilton Company, Reno, NV, USA) into the inguinal mammary fat pad of each mouse. On the sixth day after inoculation, tumor size was measured by digital caliper and ultrasound as described earlier by Danics et al. [1] (Figure 8). In the short-term experiment (3× mEHT), measurements were made at the sixth day after inoculation and at the day of termination, while in the long-term experiment (5× mEHT) tumor size was measured on every other day beginning on day six after inoculation until the termination of the experiment. Animals were randomized into mEHT-treated and sham-treated groups according to tumor size (Figure S3). Tumors were treated three or five times. Tumors were removed 24 h after the last treatment. Multiplex analyses were performed after three treatments, whereas long-term effects of repeated treatment were investigated after five treatments. Mice were euthanized by cervical dislocation, tumors were resected, cleaned and precisely cut in half along the longest diameter. One half was fixed in 4% formaldehyde solution (Molar Chemicals Ltd., Halásztelek, Hungary) and transferred for histological processing. The other half was stored in liquid nitrogen for molecular analysis (RNA isolation). Interventions and housing of the animals conformed to the Hungarian Laws No. XXVIII/1998 and LXVII/2002 about the protection and welfare of animals, and the directives of the European Union. All animal procedures were approved by the National Scientific Ethical Committee on Animal Experimentation under the No. PE/EA/50-2/2019, date of approval: 01 November 2019.

### 4.2. In Vivo Treatments

**Figure 9:**
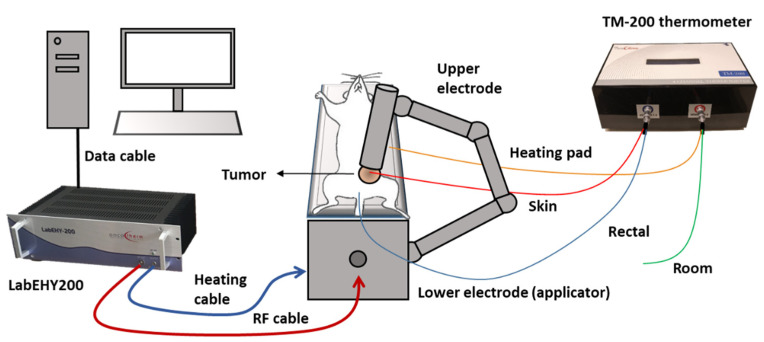
Schematic illustration of mEHT treatment setup. Mice were placed on the lower electrode (in vivo applicator) in isoflurane anesthesia. The upper electrode was positioned on the tumor in the inguinal region. The LabEHY200 was connected with the lower electrode with a radiofrequency (RF) and a heating cable. Temperature monitoring of tumor surface (red: skin temp. sensor), rectum (blue), heating pad (yellow) and room temperature (green) was performed by a TM-200 thermometer and the data were registered with a computer during the treatment.

Tumors were treated 3–5 times with the newly developed rodent modulated electro hyperthermia device as described in detail in our previous paper [1]. The principle of the treatment is a capacitively coupled, amplitude-modulated, 13.56 MHz electromagnetic field which transfers energy to the tumors. The electromagnetic field was established between two electrodes in the inguinal region. The mobile (upper) electrode was a 2 mm diameter column-shaped plastic case filled with stainless steel rods, covered with 3.1 cm^2^ silver-plated textile, positioned on the tumor. Animals were placed on a heating pad (in vivo applicator), functioning as the lower electrode, and connected to the LabEHY modulated electro hyperthermia 200 device with heating and radiofrequency (RF) cable. The abdominal area below the mobile electrode and the back of the mice was shaved before the treatments to enable electric coupling. Treatments were performed with a LabEHY 200 device in a temperature-driven way, for 30 min with 0.7 ± 0.3 watts after a 5-min-long warmup. Temperature monitoring was performed with a four-channel TM-200 thermometer (Oncotherm Ltd., Budaörs, Hungary). Temperature sensors were placed (1) on the skin above tumor, (2) in the rectum for core temperature monitoring, (3) on the heating pad and (4) nearby the treatment setup for room temperature monitoring. Skin temperature was kept at 40 ± 0.5 °C during the treatments, as it assured the required 42 °C inside the tumor. Rectal temperature was kept in the physiologic range (37.5 ± 0.5 °C), and the lower electrode’s temperature was set at the same temperature. Room temperature was at 25 ± 1 °C. During sham treatments, the electromagnetic field was turned off but all other conditions (heat pad temperature, upper electrode position) were similar to the mEHT treatment. A schematic illustration of the treatment procedure is presented in Figure 9. Numbers of animals in the three-time treatment experiments were *n*_sham_ = 7, *n*_Meht_ = 18, and *n*_sham_ = 9, *n*_mEHT_ = 7 in the five-time treatment experiment.

As demonstrated in the H&E and cC3 stained sections, five treatments had such a strong effect on the tumors that RNA isolation was troublesome, and we were not able to isolate sufficient quality RNA or protein from tumors treated three times. Thus, mRNA and proteomic studies were performed after three mEHT treatments, when tumor size reduction was already significant, but RNA and protein isolations were still possible.

### 4.3. In Vitro Treatments

In vitro treatments were performed as described by us earlier [1]. Briefly, 1 × 10^6^ 4T1 cells were pretreated in cell culture with 5 µM KRIBB11 (#385570, Sigma-Aldrich Co., St. Louis, MO, USA) or 0.01% DMSO (#D2438, Sigma-Aldrich Co., St. Louis, MO, USA) for 1h before mEHT. The cell suspension was transferred into a plastic bag for treatment with the LabEHY 200 in vitro applicator. A thermosensor (TM-200 thermometer, Oncotherm Ltd., Budaörs, Hungary) was inserted into the bag for temperature follow-up. The unit was placed in glass cuvette (filled with distilled water), which was inserted between the two electrodes of the in vitro applicator (Oncotherm Ltd., Budaörs, Hungary). An average of 4 ± 1 Watts was applied with the same amplitude-modulation (AM) as in the in vivo experiments. The temperature rise of the cell suspension was around 2.3 ± 0.8 °C/min. Cells were treated for 30 min in a temperature-driven way to maintain 42 °C in the cell suspension. Cells were collected 2 h after mEHT treatment, lysed with Tri-Reagent (#TR118/200, Molecular Research Center, Inc., Cincinnati, OH, USA) and processed for RNA isolation.

### 4.4. Histopathology and Immunohistochemistry

Formalin-fixed tumor samples were dehydrated and embedded in paraffin. Serial sections (2.5 µm) were cut for hematoxylin-eosin (H&E) staining or dewaxed and rehydrated for immunohistochemistry (IHC) using a polymer-peroxidase system (Histols, Histopathology Ltd., Pécs, Hungary). Evaluation of Tumor Destruction Ratio (TDR%) on H&E and cC3 and digital evaluation of Hsp70 and Ki67 stainings was performed as described earlier [1]. The antibodies used are listed in Table 3.

### 4.5. RNA Isolation and RT-PCR 

RNA isolation was performed with TRI reagent (Molecular Research Center Inc., Cincinatti, OH, USA) according to the manufacturer’s instructions. Isolated RNA was reverse transcribed by a High-Capacity cDNA Reverse Transcription Kit (Applied Biosystems, Carlsbadm, CA, USA). The amplified cDNA was used as a template for RT-PCR. Messenger RNAs were detected in the samples by SYBER Green based RT-PCR with SsoAdvanced™ Universal SYBER^®^ Green Supermix and the CFX96 Touch Real-Time PCR Detection System (Bio Rad, Hercules, CA, USA). Expressions were normalized to 18S. The primers used are listed in Table 4.

### 4.6. Next-Generation Sequencing and Bioinformatic Analysis

Five sham and five mEHT-treated samples were chosen based on the quality and quantity of the isolated RNA and the relative Hsp70 expression (used as a marker of effective treatment), measured by immunohistochemistry. RNA integrity and RNA concentration were assessed by the RNA ScreenTape system with the 2200 Tapestation (Agilent Technologies, Santa Clara, CA, USA) and the RNA HS Assay Kit with the Qubit 3.0 Fluorometer (Thermo Fisher Scientific, Waltham, MA, USA). The DNaseI treatment (Thermo Fisher Scientific, Waltham, MA, USA), the Ribo-Zero rRNA removal (Illumina, San Diego, CA, USA) and the KAPA Stranded RNA-Seq libraries (Roche Diagnostics, Indianapolis, IN, USA) were prepared according to manufacturer’s protocols. The quality and quantity of the libraries were determined by the High Sensitivity DNA1000 ScreenTape system with the 2200 Tapestation (Agilent Technologies, Santa Clara, CA, USA) and dsDNA HS Assay Kits with Qubit 3.0 Fluorometer (Thermo Fisher Scientific, Waltham, MA, USA). Pooled libraries were diluted to 1.6 pM for 2 × 80 bp paired-end sequencing with 150-cycles of the High Output v2 Kit on the NextSeq 550 Sequencing System (Illumina, San Diego, CA, USA) according to the manufacturer’s protocol. Raw sequenced reads >50 M per sample were demultiplexed and adapter-trimmed by using the NextSeq Control Software, whilst the FastQ Toolkit (Illumina, San Diego, CA, USA) was applied to trim bases at the 3′- and the 5′-ends with a quality score <30. Reads with mean quality score <30 and shorter than 32 bp were filtered out.

Reads were compared with the Mus musculus reference genome (GRCm38 Ensembl release, STAR v2.6.1c) [80]. After alignment, the reads were associated with known protein-coding genes and the number of reads aligned within each gene was counted using the HTSeq tool v0.6.1p1 [81]. Gene count data were normalized using the trimmed mean of M values (TMM) normalization method of the edgeR R/Bioconductor package (v3.28, R v3.6.0, Bioconductor v3.9) [82]. For statistical testing, the data were further log transformed using the voom approach [83] in the limma package [84]. TMM normalized counts were represented as transcripts per million (TPM) values. Fold-change (FC) values between the compared groups, resulting from a linear modeling process and modified t-test *p*-values, were produced by the limma package. FC > 2.0 and *p*-value < 0.05 thresholds were used for filtering the differentially expressed (DE) genes. Based on intensive literature search on PubMed, UniProt, Protein Atlas, nonprotein-coding genes, previously unidentified (no literature search possible) genes and those that had no/insufficient literature (lack of information on expression, function, and regulation) were excluded from further analysis. Remaining DE genes were grouped into functional categories created by us. Functional analysis was performed to consider the functional annotations of genes using the gene ontology (GO) database. Detection of functional enrichment was performed in the differentially expressed gene list (DE list enrichment) and towards the top of the list when all genes were ranked according to the evidence for being differentially expressed (ranked list enrichment) applying the topGO v2.37.0 packages. We show results of the GO analysis of significant upregulated genes (DEListEnrichment_upR). A heat map was created from the normalized NGS RNA Seq data with Kendall tau’s method at heatmapper.ca (Wishart Research Group, University of Alberta, Canada) [85]. Raw RNA-Seq data sets generated as part of this study will be publicly available at the European Nucleotide Archive (https://www.ebi.ac.uk/ena, accessed on 19 March 2021), under study accession number PRJEB43813.

### 4.7. Mass Spectrometry Analysis

Mass spectrometry analysis of the same samples used for NGS was performed as described earlier by Róka et al. [19]. Briefly, liquid chromatography with tandem mass spectrometry (LC-MS/MS) analysis was done using an EASY-nanoLC II HPLC unit (Thermo Fisher Scientific, Waltham, MA, USA) coupled with an Orbitrap LTQ Velos mass spectrometer (Thermo Fisher Scientific, Waltham, MA, USA). Samples containing 0.1% FA were loaded onto a C18 trapping column (Proxeon Easy-column, Thermo Fischer Scientific, West Palm Beach, FL, USA) and separated on a C18 PicoFrit Aquasil analytical column (New Objective, Inc., Woburn, MA, USA). The peptides were eluted using a 5–40% (*v*/*v*) 90 min linear gradient of acetonitrile in a 0.1% formic acid solution at a constant flow rate of 300 nL/min. The full MS mass spectra were acquired with the Orbitrap mass analyzer in the mass range of 300 to 2000 *m*/*z* at a resolution of 30,000. The MS/MS spectra were obtained by higher-energy collisional dissociation (HCD) fragmentation of the nine most intense MS precursor ions and recorded at a resolution of 7500. Only the precursor ions with assigned charge states (>1) were selected for MS/MS fragmentation. The dynamic exclusion was set to a repeat count of 1, repeat duration of 30 s, and exclusion duration of 20 s. For data analysis, the MaxQuant proteomics software (version 1.6.0.13; Max-Planck Institute for Biochemistry, Martinsried, Germany) was used for database search and quantification by spectral counting [86]. The database search was performed against a Mus musculus Uniprot database (database date 15.10.2017, 16,923 entries). For the database searches, methionine oxidation (+15995 Da) and protein N-terminal acetylation (+45011 Da) were set as variable modifications. Carbamidomethylation of cysteines (+57021 Da) was set as a fixed modification. Trypsin cleavage at arginine and lysine residues was used as enzyme specificity. For the database search, one missed cleavage was allowed. In addition, precursor ion and fragment ion mass tolerances were set to 20 ppm and 0.5 Da, respectively. A reversed database search was performed, and the false discovery rate was set to 1% for peptide and protein identifications. Raw data and database search files are available at ProteomeXchange (identifier PXD024150) [87]. Relative quantification of identified proteins was performed by label-free quantification (LFQ) algorithm in MaxQuant.

### 4.8. Nanostring Analysis

RNA samples from the same tumors used for sequencing (NGS), and two further samples per group, were chosen for gene expression validation by nanostring. RNA concentrations measured by a Qubit 4 Fluorometer (Thermo Fisher Scientific, USA). RNA samples with adequate concentrations were hybridized to the customized nCounter^®^ gene panel (NanoString, Redwood, CA, USA). The applied custom gene panel was composed of 134 genes identified by NGS as differentially expressed with the highest FC and lowest *p* values. Genes with no or deficient information according to the literature were excluded. Samples were transferred to the nCounter Prep Station for further processing. The gene expression profiles of the samples were digitized with the nCounter Digital Analyzer. Results were quantified using the nSolver 4.0 Analysis Software (NanoString, Redwood, CA, USA). Background was determined with synthetic negative probes provided by the Nanostring company, calculating the background level at maximum negative control count number.

### 4.9. Statistical Analysis

GraphPad Prism software (v.6.01; GraphPad Software, Inc., La Jolla, CA, USA) was applied for statistical analysis. Unpaired Mann-Whitney nonparametric tests were performed in the comparison of sham and mEHT treated groups. Follow-up examinations were statistically evaluated with two-way ANOVA with Bonferroni correction. Differences were considered statistically significant as * *p* < 0.05, ** *p* < 0.01, *** *p* < 0.001, **** *p* < 0.0001. Data are presented as mean ± SEM.

## 5. Conclusions

Here, we demonstrated that modulated electro-hyperthermia (mEHT) effectively inhibited tumor growth and proliferation. Moreover, mEHT activated several stress response genes such as members of the heat shock response, complement factors such as C4, fibrinogens, haptoglobin and pentraxin, locally in the tumor. Applying KRIBB11 + mEHT in combination may have a synergistic effect in vivo, potentiating mEHT’s antitumor effects. Therefore this will be the focus of our future work. Inhibition of these protective mechanisms has the potential to enhance the effectivity of anticancer therapies, including mEHT and other clinically applied, traditional treatment modalities like chemo, radio and immunotherapy.

## Figures and Tables

**Table 1:** Absolute mRNA Count of cellular stress response factors from the Nanostring data. Individual data of sham and mEHT group members and group averages. **Bold letters**: genes with highest mRNA absolute count and fold-change between the two groups. Background values measured in negative, synthetic probe RNA counts were between 0–16. Cell line: 4T1, 3× treated.

RNA Count	Sham							mEHT						
Genes	#5	#6	#11	#14	#16	#26	Avg.	#4	#10	#12	#15	#20	#27	Avg.
Itih2	2	3	6	9	1	2	3.8	7	54	63	7	27	27	30.8
Itih4	48	9	9	13	2	4	14.2	196	42	60	20	158	40	86.0
**Serpina3n**	**109**	**62**	**186**	**110**	**25**	**96**	**98.0**	**1265**	**614**	**565**	**444**	**1016**	**352**	**709.3**
Serpina3c	69	37	78	47	12	18	43.5	284	191	216	64	251	111	186.2
Serpina3m	17	13	16	15	7	8	12.7	85	49	30	19	75	34	48.7
Fgb	2	7	21	3	4	5	7.0	7	278	116	18	61	118	99.7
Fgg	7	16	9	19	4	5	10.0	4	103	121	18	19	170	72.5
**Hp**	**7782**	**3082**	**4825**	**7449**	**1505**	**1632**	**4379.2**	**48,983**	**25,954**	**16,208**	**11,680**	**56,174**	**16,738**	**29,289.5**
**Ptx3**	**244**	**87**	**180**	**100**	**15**	**107**	**122.2**	**2024**	**568**	**983**	**566**	**2202**	**185**	**1088.0**
**Cfd**	**3224**	**975**	**1846**	**2293**	**260**	**402**	**1500.0**	**22,431**	**7571**	**7408**	**2341**	**16,311**	**4961**	**10,170.5**
**C4b**	**1242**	**1081**	**997**	**1244**	**166**	**1443**	**1028.8**	**9234**	**4573**	**3806**	**2930**	**5258**	**1794**	**4599.2**
Hc	17	28	16	32	6	33	22.0	2	77	47	6	6	49	31.2
C1s1	493	479	744	233	141	345	405.8	2036	1638	1390	841	979	672	1259.3

**Table 2:** Cellular stress response upregulated by mEHT treatment. Genes (official name abbreviations as used in all multiplex platforms and descriptions of the coded proteins) detected as upregulated with all three multiplex methods (NGS RNA Seq, Nanostring, MS) are designated with **bold** letters. Nanostring validated all NGS hits with a similar FC value. Hspa1a and Hspa1b are the most common isoforms of Hsp70. Cell line: 4T1, 3× treated.

Nr.	Gene Name	Description	NGS		Nanostring		MS	
			FC	*p*	FC	*p*	LFQ Intensity Difference	*p*
**Protease inhibitors**								
1	Itih2	inter-alpha trypsin inhibitor. heavy chain 2	31.1	2.1 × 10^−5^	*		2.9	7.7 × 10^−5^
2	**Itih4**	**inter alpha-trypsin inhibitor. heavy chain 4**	**22.7**	**2.7 × 10^−4^**	**9.5**	**0.005**	**2.5**	6.1 × 10^−5^
3	**Serpina3n**	**serine (or cysteine) peptidase inhibitor. clade A. member 3N**	**8.4**	**6.8 × 10^−4^**	**8.1**	**9.11 × 10^−5^**	**3.2**	6.1 × 10^−4^
4	Serpina3c	serine (or cysteine) peptidase inhibitor. clade A. member 3C	7.0	0.011	5.3	5.0 × 10^−4^	Not detected	Not detected
5	Serpina3m	serine (or cysteine) peptidase inhibitor. clade A. member 3M	6.4	0.001	6.2	6.1 × 10^−5^	0.1	0.891
**Coagulation factors**								
6	**Fgb**	**fibrinogen beta chain**	**28.4**	**2.1 × 10^−4^**	*		**2.4**	5.2 × 10^−6^
7	**Fgg**	**fibrinogen gamma chain**	**15.0**	**0.006**	**14.2**	**0.003**	**2.1**	3.8 × 10^−5^
8	**Hp**	**haptoglobin**	**9.7**	**7.4 × 10^−4^**	**7.5**	**2.5 × 10^−4^**	**4.7**	8.3 × 10^−4^
**Complement factors**								
9	Ptx3	pentraxin related gene	5.6	0.046	10.1	7.5 × 10^−4^	Not detected	Not detected
10	**Cfd**	**complement factor D (adipsin)**	**10.0**	**0.001**	**7.8**	**8.8 × 10^−4^**	**2.0**	0.001
11	**C4b**	**complement component 4B (Chido blood group)**	**4.6**	**0.03**	**4.8**	**0.001**	**3.1**	2.9 × 10^−4^
12	Hc	hemolytic complement	3.9	0.04	1.9	0.335	1.2	1.7 × 10^−4^
13	C1s1	complement component 1. s subcomponent 1	3.4	0.049	3.3	0.002	Not detected	Not detected
**Heat shock factors**								
	Hspb1	Heat shock protein beta-1	3.8	0.075	not investigated		2.7	1.1 × 10^−5^
	Hspa1a	Heat shock 70 kDa protein 1A	2.0	0.551	not investigated		2.1	1.3 × 10^−5^
	Hspa1b	Heat shock 70 kDa protein 1B	2.4	0.362				
	Hsph1	Heat shock protein 105 kDa	1.8	0.761	not investigated		1.3	0.023

* uniquely expressed in mEHT-treated samples but not in the sham-treated tumors, no FC applies by the nanostring evaluation.

**Table 3:** Antibodies and conditions used for immunohistochemistry and immunofluorescence. pAb: polclonal antibody, Hsp70: heat shock protein-70, Ki67: marker of proliferation.

Antigen	Type	Reference No.	Dilution	Vendor ^1^
Hsp70	Rabbit, pAb	#4872	1:200	Cell Signaling
Ki67	Rabbit, pAb	#RM-9106	1:400	Thermo

^1^ Vendor specifications: Cell Signaling (Danvers, MA, USA), Thermo (Waltham, MA, USA).

**Table 4:** Primers used for RT-PCR.

Gene Symbol	Gene Name	Primer Pairs
18S	18S	Fwd: CTCAACACGGGAAACCTCAC
	[Mus musculus]	Rev: CGCTCCACCAACTAAGAACG
C4b	Complement component 4b	Fwd: AACCCCTCGACATGAGCAAG
	[Mus musculus]	Rev: TGGAACACCTGAAGGGCATC

## Data Availability

Data available in a publicly accessible repository that does not issue DOIs. Publicly available datasets were analyzed in this study. This data can be found here: NGS: https://www.ebi.ac.uk/ena, accession number: PRJEB43813; MS: http://www.proteomexchange.org/, identifier: PXD024150.

## Supplementary Materials

The following are available online at https://www.mdpi.com/article/10.3390/cancers13071744/s1, Figure S1. Hematoxylin-eosin (HE) and cleaved caspase-3 (cC3) immunohistochemistry stained sections of all tumors by groups, 24 h after the fifth mEHT treatment. Figure S2. Heat map dendogram of the differentially expressed (DE) genes with labels after 3 mEHT treatments. Figure S3. Tumor volumes measured by ultrasound at randomization (day 6 after inoculation). Table S1. Upregulated genes in the response to stimulus pathway as identified by the gene ontology (GO) analysis. Table S2. Upregulated genes in stress response related pathways as identified by the gene ontology (GO) analysis.

## Abbreviations

APPs	Acute phase proteins
C4	Complement component 4
cC3	Cleaved caspase-3
DE	Differentially expressed
EMF	Electromagnetic field
FC	Fold change
H&E	Hematoxylin and eosin
HER2	Human epidermal receptor growth factor receptor 2
Hsp70	Heat shock protein 70
IHC	Immunohistochemistry
LFQ	Label-free quantification
mEHT	Modulated electro-hyperthermia
mRNA	Messenger ribonucleic acid
MS	Mass spectrometry
NGS RNA seq	Next-generation sequencing ribonucleic acid sequencing
RF	Radio frequency
RT-qPCR	Real-time quantitative polymerase chain reaction
TDR	Tissue damage ratio
TNBC	Triple-negative breast cancer
