# Supplementary material for: Modulated Electro-Hyperthermia Induces a Prominent Local Stress Response and Growth Inhibition in Mouse Breast Cancer Isografts
Source: Cancers (Basel). 2021 Apr 6;13(7):1744. doi: 10.3390/cancers13071744 (PMC8038813; doi:10.3390/cancers13071744)
Supplement: Supplementary file 1 [file cancers-13-01744-s001.pdf]

cancers MDPI

Supplementary Materials: Modulated Electro-Hyperthermia Induces a Prominent Local Stress Response and Growth Inhibition in Mouse Breast Cancer Isografts

Csaba András Schvarcz, Lea Danics, Tibor Krenács, Pedro Leroy Viana, Rita Béres, Tamás Vancsik, Ákos Nagy, Attila Gyenesei, József Kun, Marko Fonović, Robert Vidmar, Zoltán Benyó, Tamás Kaucsár and Péter Hamar

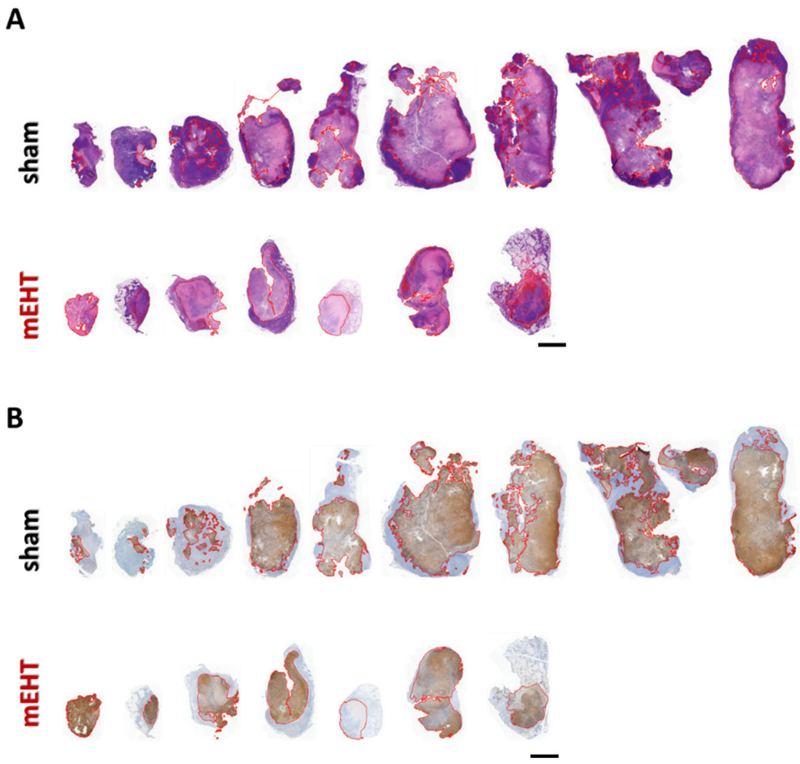

Figure S1. Hematoxylin-eosin (HE) (A) and cleaved caspase-3 (cC3) (B) immunohistochemistry stained sections of all tumors by groups, 24h after the fifth mEHT treatment. Destructed area is annotated (red) for calculation of TDR. ($n_{(sham)}$ = 9, $n_{(mEHT)}$ =7. Scale bar: 2000 µm).

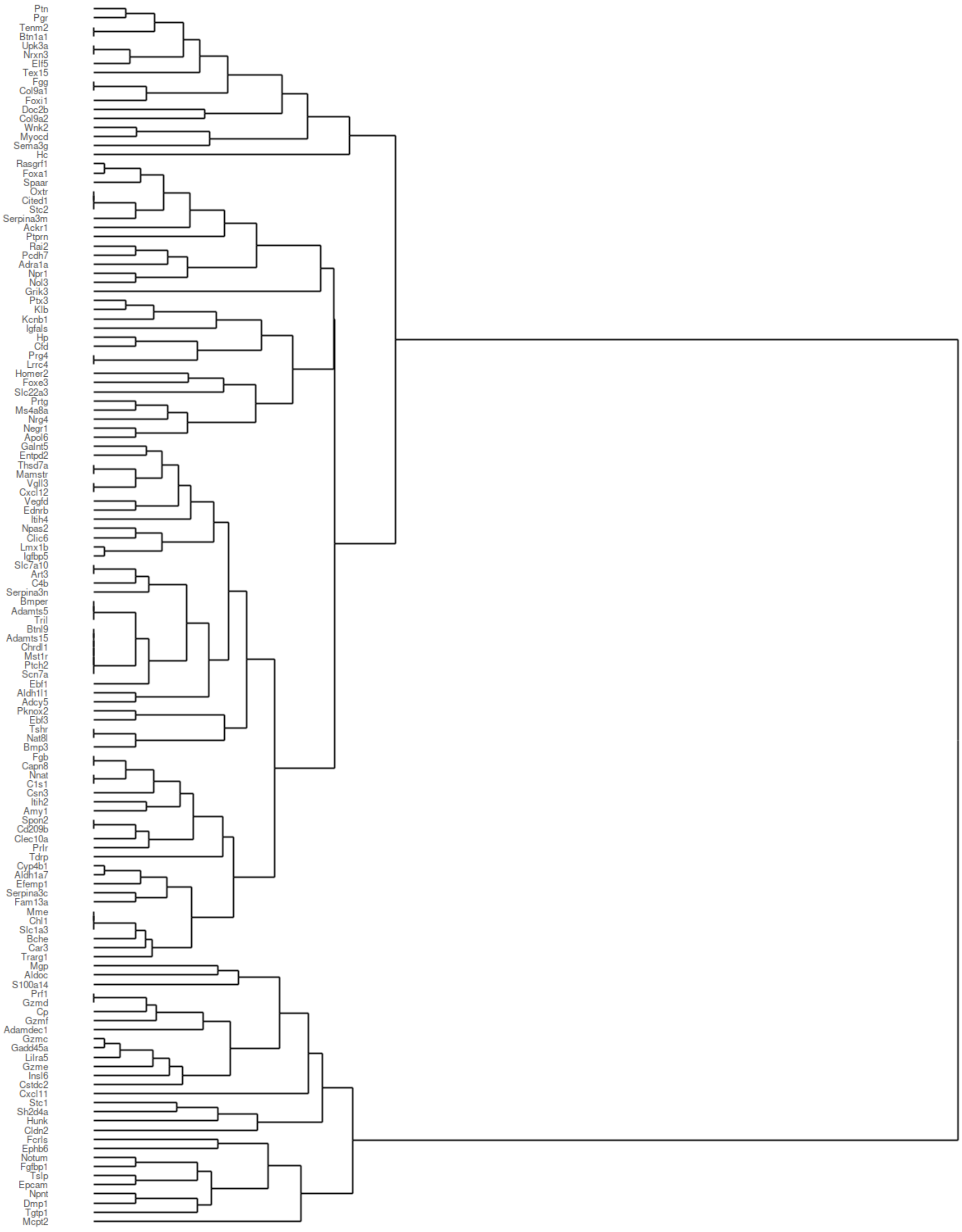

Figure S2. Heat map dendogram of the differentially expressed (DE) genes with labels after 3 mEHT treatments.

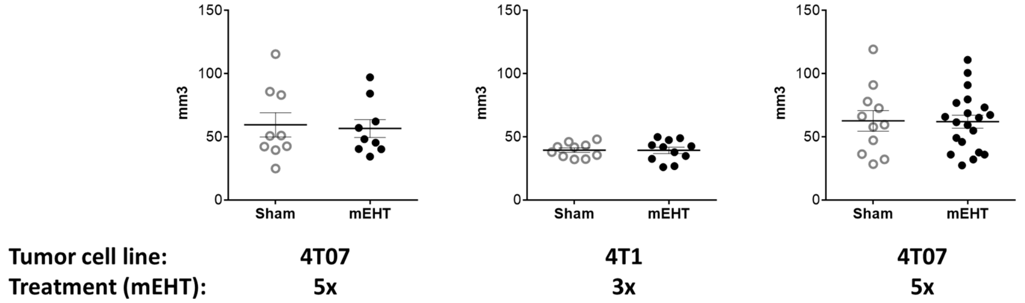

Figure S3. Tumor volumes measured by ultrasound at randomization (day 6 after inoculation). Treatment interval: 48h, harvest: 24h after last treatment.

Table S1. Tabular display of Figure 6.: upregulated genes in the response to stimulus pathway as identified by the gene ontology (GO) analysis.

Gene Name	Description	*p*	FC
Fgb	fibrinogen beta chain	2.1E-04	28.4
Itih4	inter alpha-trypsin inhibitor, heavy chain 4	2.7E-04	22.7
Klb	klotho beta	1.5E-03	17.7
Car3	carbonic anhydrase 3	3.5E-04	16.9
Nnat	neuronati	2.3E-03	15.8
Bmper	BMP-binding endothelial regulator	4.3E-03	15.6
Fgg	fibrinogen gamma chain	5.9E-03	15.0
Prlr	prolactin receptor	9.3E-04	14.9
Slc7a10	solute carrier family 7 (cationic amino acid transporter, y+ system), member 10	2.0E-03	13.6
Cited1	Cbp/p300-interacting transactivator with Glu/Asp-rich carboxy-terminal domain 1	1.3E-04	12.9
Foxa1	forkhead box A1	2.8E-03	12.0
Oxtr	oxytocin receptor	5.7E-04	11.5
Tshr	thyroid stimulating hormone receptor	4.4E-03	11.5
Bche	butyrylcholinesterase	1.8E-03	11.3
Igfals	insulin-like growth factor binding protein, acid labile subunit	4.7E-03	11.0
Gpr182	G protein-coupled receptor 182	8.0E-04	10.3
Cfd	complement factor D (adipsin)	1.2E-03	10.0
Amy1	amylase 1, salivary	2.1E-03	9.7
Hp	haptoglobin	7.4E-04	9.7
Lmx1b	LIM homeobox transcription factor 1 beta	4.0E-03	9.5
Slc36a2	solute carrier family 36 (proton/amino acid symporter), member 2	1.8E-03	9.3
Nat8l	N-acetyltransferase 8-like	7.8E-03	9.2
Grik3	glutamate receptor, ionotropic, kainate 3	1.0E-03	8.4
Slc1a3	solute carrier family 1 (glial high affinity glutamate transporter), member 3	3.3E-03	8.4
Serpina3n	serine (or cysteine) peptidase inhibitor, clade A, member 3N	6.8E-04	8.4
Slc22a3	solute carrier family 22 (organic cation transporter), member 3	1.0E-03	7.5
Rasgrf1	RAS protein-specific guanine nucleotide-releasing factor 1	6.3E-03	7.4
Igfbp5	insulin-like growth factor binding protein 5	2.8E-03	6.9
Cxcl12	chemokine (C-X-C motif) ligand 12	5.4E-03	6.8
Slc25a23	solute carrier family 25 (mitochondrial carrier; phosphate carrier), member 23	4.3E-03	6.7
Dpyd	dihydropyrimidine dehydrogenase	6.9E-03	6.5
Serpina3m	serine (or cysteine) peptidase inhibitor, clade A, member 3M	1.1E-03	6.4
Stc2	stanniocalcin 2	3.2E-03	6.1
Trarg1	trafficking regulator of GLUT4 (SLC2A4) 1	8.9E-03	5.3
Adamts3	a disintegrin-like and metallopeptidase (reprolysin type) with thrombospondin type 1 motif, 3	3.7E-03	5.2
Draxin	dorsal inhibitory axon guidance protein	6.6E-03	5.1
Cyp4b1	cytochrome P450, family 4, subfamily b, polypeptide 1	7.1E-03	5.1
Zfp423	zinc finger protein 423	4.8E-03	3.9
	**response to stimulus, GO:0050896**		

**p = 0.00012, genes: 38**

Top row: GO identification number and p-value of the pathway, and the number of genes upregulated by modulated electro-hyperthermia (mEHT) in the pathway. Table: gene descriptions, and individual p and FC values of the genes.

Table S2. Upregulated genes in stress response related pathways as identified by the gene ontology (GO) analysis.

Gene Name	p	FC	Gene Name	p	FC	Gene Name	p	FC	Gene Name	p	FC
Fgb	2.1E-04	28.4	Itih2	2.1E-05	31.1	Ptx3	4.6E-02	5.6	Fgb	2.1E-04	28.4
Cd5l	1.6E-01	19.6	Itih4	2.7E-04	22.7	C4b	3.0E-02	4.6	Bmper	4.3E-03	15.6
Spon2	1.3E-02	15.5	Serpina3n	6.8E-04	8.4	Vsig4	2.0E-01	3.5	Fgg	5.9E-03	15.0
Cfd	1.2E-03	10.0	Serpina3c	1.1E-02	7.0	Serping1	1.1E-01	2.6	Dmbt1	5.1E-02	12.1
Reg3g	1.3E-01	4.6	Serpina3m	1.1E-03	6.4	Cd93	3.0E-01	2.3	Col9a2	2.5E-03	11.6
C4b	3.0E-02	4.6	Serpinb2	2.5E-01	4.0	C5ar1	3.7E-01	2.3	Col9a1	2.9E-02	8.0
Hc	4.0E-02	3.9	Col28a1	1.2E-01	3.1	Cfh	4.6E-01	2.1	Ogn	7.9E-02	8.0
Cd37	1.1E-01	3.7	Serping1	1.1E-01	2.6	Cd59a	5.0E-01	2.0	Efemp1	3.8E-02	7.3
Ppbp	3.5E-01	3.6	Wfdc17	3.9E-01	2.4	-	-	-	Col6a6	9.9E-02	5.1
Pf4	2.1E-01	3.6	Serpina1b	3.0E-01	2.4	-	-	-	Vtn	1.1E-01	5.0
Vsig4	2.0E-01	3.5	-	-	-	-	-	-	Prelp	4.5E-02	4.0
Hpx	2.3E-01	3.5	-	-	-	-	-	-	Spock2	9.8E-02	3.9
C1s1	4.9E-02	3.4	-	-	-	-	-	-	Col17a1	9.1E-02	3.8
Ltf	2.7E-01	3.0	-	-	-	-	-	-	Matn4	1.4E-01	3.8
Cd55	2.6E-01	2.9	-	-	-	-	-	-	Col28a1	1.2E-01	3.1
Cfi	3.3E-01	2.9	-	-	-	-	-	-	Dcn	6.8E-02	3.1
Cxcl3	3.8E-01	2.7	-	-	-	-	-	-	Vcan	9.0E-02	3.1
C2	1.7E-01	2.6	-	-	-	-	-	-	Dpt	2.3E-01	3.0
Serping1	1.1E-01	2.6	-	-	-	-	-	-	Lama2	1.2E-01	2.9
Col20a1	3.4E-01	2.4	-	-	-	-	-	-	Col14a1	2.6E-01	2.7
C1rb	3.5E-01	2.3	-	-	-	-	-	-	Col3a1	1.8E-01	2.6
-	-	-	-	-	-	-	-	-	Comp	2.8E-01	2.6
-	-	-	-	-	-	-	-	-	Fbln1	1.4E-01	2.6
-	-	-	-	-	-	-	-	-	Hmcn2	2.0E-01	2.6
-	-	-	-	-	-	-	-	-	Col5a3	5.3E-02	2.5
-	-	-	-	-	-	-	-	-	Col9a3	3.3E-01	2.4
-	-	-	-	-	-	-	-	-	Fbn2	2.6E-01	2.4
-	-	-	-	-	-	-	-	-	Slit2	2.8E-01	2.4
-	-	-	-	-	-	-	-	-	Fbn1	2.5E-01	2.3
-	-	-	-	-	-	-	-	-	Col6a5	4.6E-01	2.2
-	-	-	-	-	-	-	-	-	Mmrn2	3.5E-01	2.2
-	-	-	-	-	-	-	-	-	Emilin2	3.9E-01	2.2
-	-	-	-	-	-	-	-	-	Hmcn1	4.3E-01	2.1
-	-	-	-	-	-	-	-	-	Bgn	3.8E-01	2.1
-	-	-	-	-	-	-	-	-	Col1a1	4.3E-01	2.1
-	-	-	-	-	-	-	-	-	Col15a1	4.6E-01	2.1
-	-	-	-	-	-	-	-	-	Nid1	4.8E-01	2.0
-	-	-	-	-	-	-	-	-	Col27a1	4.9E-01	2.0
-	-	-	-	-	-	-	-	-	Fras1	6.6E-01	1.7
**humoral immune response GO:0006959**			**serine-type endopeptidase inhibitor activity GO:0004867**			**complement binding GO:0001848**			**extracellular matrix structural constituent binding GO:0005201**		
**p = 1.38e-05, genes: 21**			**p = 1.9e-05, genes: 10**			**p = 0.000241, genes: 8**			**p = 4.9e-11, genes: 39**		

Top row: GO identification numbers and p-values of the pathways, and the number of genes upregulated by modulated electrohyperthermia (mEHT) in the pathways. Table: gene descriptions, and individual p and FC values of the genes.
